# Health Tract, No. 295

**Published:** 1867-08

**Authors:** 


HEALTH TRACT, No. 295.

### SMOKEY CHIMNEYS

Are an annoyance and a source of constant irritation to many households besides the discomfort, gloom, dinginess and dirt which are imparted to kitchen or parlor. Where the houses are close together the chimneys must out-top the neighboring building or wall. But there are two things to be done which will seldom fail to make any chimney draw well. The "flue" is the passage for the smoke upwards from the fire; smoke is very light, and if in its ascent it strikes against any thing, it rebounds especially if the obstacle is immediately above the fire, for there the draft is comparatively light, but it increases as it ascends. The throat of the chimney is the beginning of the chimney part, and is usually three or four inches broad or deep, and ten, fifteen or more lengthways or across; if this throat is abruptly widened and lengthened so as to make a chamber twice as large in both directions, and a foot high, and then taper off as desired, the chimney will not smoke, especially after this chamber has become somewhat heated, for the rarified air creates a vacuum into which the smoke rushes and a strong draft is immediately generated. Another item in connection with chimneys merits attention, as many houses have been set on fire and lives lost by the "chimney taking fire," as it is termed, in consequence of the great accumulation of soot in the body of the chimney. It is claimed that if salt is mixed with the mortar which is used in building the chimney in the usual way, the accumulation of soot is prevented by the salt absorbing the moisture every damp day, and parting with it to the soot, which thus becomes heavy and falls down into the fire-place.

If smoke has settled on the walls whether from chimneys or from gas or oil lights, it may be removed preparatory to whitewashing and the walls will be left in good condition to receive the lime if some indigo is dissolved in water and the mixture is applied with a brush; another plan has been suggested: take four quarts of fresh wood ashes in a wooden vessel, pour on two gallons of boiling water and when settled apply with a brush; if the first brushing thus is not sufficient let it dry and make another application. It adds so much to the feeling of comfort to have a tidy, clean, light-looking kitchen that every good housekeeper will value the suggestions made.

